# Correction: *Does tranexamic acid lead to changes in MRI measures of brain tissue health in patients with spontaneous intracerebral haemorrhage? Protocol for a MRI substudy nested within the double-blind randomised controlled TICH-2 trial*

**DOI:** 10.1136/bmjopen-2017-019930corr1

**Published:** 2018-04-20

Dineen RA, Pszczolkowski S, Flaherty K, *et al*. Does tranexamic acid lead to changes in MRI measures of brain tissue health in patients with spontaneous intracerebral haemorrhage? Protocol for a MRI substudy nested within the double-blind randomised controlled TICH-2 trial. *BMJ Open* 2018;8:e019930. doi: 10.1136/bmjopen-2017-019930

On the secondary outcomes subsection (page 3) it reads:

‘Only DWIHL that are confirmed of low diffusion on the derived apparent diffusion coefficient maps and spatially remote from the index ICH (<20 mm) will be included as previously.’

It should read:

*‘*Only DWIHL that are confirmed of low diffusion on the derived apparent diffusion coefficient maps and spatially remote from the index ICH **(>20 mm)** will be included as previously.*’*

