# Supplementary material for: Does tranexamic acid lead to changes in MRI measures of brain tissue health in patients with spontaneous intracerebral haemorrhage? Protocol for a MRI substudy nested within the double-blind randomised controlled TICH-2 trial
Source: BMJ Open. 2018 Feb 3;8(2):e019930. doi: 10.1136/bmjopen-2017-019930 (PMC5879748; doi:10.1136/bmjopen-2017-019930)
Supplement: Supplementary file 1 [file bmjopen-2017-019930supp001.pdf]

Supplementary information: Brief summary of the TICH-2 Trial design with list of inclusion and exclusion criteria*

TICH-2 is a phase III prospective double-blind randomized placebo-controlled trial that aims to test the hypothesis that intravenous tranexamic acid given within 8 h of SICH reduces death or dependency. 2000 participants within 8 hours of SICH will be randomized to receive either intravenous tranexamic acid 1 g 10 min bolus followed by 1 g 8 h infusion, or placebo. The primary outcome is death or dependency measured by ordinal shift analysis of the 7 level mRS at
day 90. Secondary outcomes are neurological impairment at day 7 and disability, quality of life, cognition, and mood at day 90. Safety outcomes are death, serious adverse events, thromboembolic events, and seizures. Cost outcomes are length of stay in hospital, readmission, and institutionalization.

Inclusion criteria:

- Adults with acute SICH within 8 h of stroke symptom onset or time last seen well.

Exclusion criteria:

- Patients with ICH secondary to anticoagulation, thrombolysis or known underlying structural abnormality such as arteriovenous malformation, aneurysm, tumour, or venous thrombosis. An underlying structural abnormality does not need to be excluded before enrolment, but where known, patients should not be recruited.
- Contraindication to tranexamic acid.
- Premorbid dependency (mRS>4).
- Concurrent participation in another drug or device trial. Participants enrolled in TICH-2 may be enrolled into the RESTART trial18 after 21 days.
- Pre-stroke life expectancy <3 months (e.g. advanced metastatic cancer).
- Coma—Glasgow coma scale <5.
- ICH was secondary to trauma.
- Women of childbearing potential, pregnant, or breastfeeding at randomization.
- Geographical or other factors that prohibit follow-up at 90 days, e.g. no fixed address or telephone contact number, or overseas visitor.

* Sprigg N, Robson K, Bath PM, *et al.* Intravenous tranexamic acid for hyperacute primary intracerebral hemorrhage: Protocol for a randomized, placebo-controlled trial. *Int J stroke* 2016;**11**:717–23
