# Supplementary material for: Does tranexamic acid lead to changes in MRI measures of brain tissue health in patients with spontaneous intracerebral haemorrhage? Protocol for a MRI substudy nested within the double-blind randomised controlled TICH-2 trial
Source: BMJ Open. 2018 Feb 3;8(2):e019930. doi: 10.1136/bmjopen-2017-019930 (PMC5879748; doi:10.1136/bmjopen-2017-019930)
Supplement: Author's manuscript [file bmjopen-2017-019930.draft_revisions.pdf]

BMJ Open is committed to open peer review. As part of this commitment we make the peer review history of every article we publish publicly available.

When an article is published we post the peer reviewers' comments and the authors' responses online. We also post the versions of the paper that were used during peer review. These are the versions that the peer review comments apply to.

The versions of the paper that follow are the versions that were submitted during the peer review process. They are not the versions of record or the final published versions. They should not be cited or distributed as the published version of this manuscript.

BMJ Open is an open access journal and the full, final, typeset and author-corrected version of record of the manuscript is available on our site with no access controls, subscription charges or pay-per-view fees (http://bmjopen.bmj.com).

If you have any questions on BMJ Open's open peer review process please email editorial.bmjopen@bmj.com

BMJ Open

Rationale and Design of Tranexamic acid for hyperacute primary IntraCerebral Haemorrhage (TICH-2) MRI substudy

Journal:	*BMJ Open*
Manuscript ID	bmjopen-2017-019930
Article Type:	Protocol
Date Submitted by the Author:	03-Oct-2017
Complete List of Authors:	Dineen, Rob; University of Nottingham School of Medicine, Division of Clinical Neuroscience<br>Pszczolkowski, Stefan; University of Nottingham School of Medicine, Division of Clinical Neuroscience<br>Flaherty, Katie; University of Nottingham School of Medicine, Division of Clinical Neuroscience<br>Law, Zhe; University of Nottingham School of Medicine, Division of Clinical Neuroscience<br>Morgan, Paul; University of Nottingham School of Medicine, Division of Clinical Neuroscience<br>Roberts, Ian; London School of Hygiene and Tropical Medicine, Epidemiology and Population Health<br>Werring, David; University College London, Stroke Research Centre<br>Salman, Rustam; University of Edinburgh, Centre for Clinical Brain Sciences<br>England, Tim; University of Nottingham School of Medicine, Vascular Medicine, Division of Medical Sciences & GEM<br>Bath, Philip; University of Nottingham School of Medicine, Division of Clinical Neuroscience<br>Sprigg, Nicola; University of Nottingham School of Medicine, Division of Clinical Neuroscience
Keywords:	hyperacute primary intracerebral haemorrhage, tranexamic acid, Magnetic resonance imaging < RADIOLOGY & IMAGING, diffusion weighted imaging, perihaematomal oedema

SCHOLARONE™
Manuscripts

Title: Rationale and Design of Tranexamic acid for hyperacute primary IntraCerebral Haemorrhage (TICH-2) MRI substudy

Authors:

Rob A. Dineen[1,2,3], Stefan Pszczolkowski[1,4], Katie Flaherty[4], Zhe K. Law[4,5], Paul S. Morgan[1,2,6], Ian Roberts[7], David Werring[8], Rustam Al-Shahi Salman[9], Tim England[10], Philip M. Bath[4], Nikola Sprigg[4]

[1]Radiological Sciences, Division of Clinical Neuroscience, University of Nottingham, Nottingham, UK

[2]Sir Peter Mansfield Imaging Centre, University of Nottingham, Nottingham, UK

[3]NIHR Nottingham Biomedical Research Centre, Nottingham, UK

[4]Stroke Trials Unit, Division of Clinical Neuroscience, University of Nottingham, Nottingham, UK

[5]National University of Malaysia, Kuala Lumpur, Malaysia

[6]Medical Physics and Clinical Engineering, Nottingham University Hospitals NHS Trust, Nottingham, UK

[7]London School of Hygiene & Tropical Medicine, London, UK

[8]Stroke Research Centre, University College London, London, UK

[9]Centre for Clinical Brain Sciences, University of Edinburgh, Edinburgh, UK

[10]Vascular Medicine, Division of Medical Sciences & GEM, University of Nottingham, Nottingham, UK

Correspondence: Dr. Rob Dineen, Division of Clinical Neuroscience, University of Nottingham, Queen's Medical Centre, Derby Road, Nottingham NG7 2UH, UK.

Email rob.dineen@nottingham.ac.uk

Key words:

For peer review only

hyperacute primary intracerebral haemorrhage, tranexamic acid, magnetic resonance imaging, diffusion weighted imaging, perihaematomal oedema

Word count

2242 words for main text

For peer review only

ABSTRACT

Objectives: To test whether administration of the antifibrinolytic drug tranexamic acid (TXA) in patients with spontaneous intracerebral haemorrhage (SICH) leads to increased prevalence of diffusion-weighted MRI defined hyperintense ischaemic lesions (primary hypothesis) or reduced perihaematomal oedema volume, perihaematomal diffusion restriction and residual MRI-defined SICH-related tissue damage (secondary hypotheses).

Design: MRI sub-study nested within the double-blind randomised controlled TICH-2 trial (ISRCTN93732214).

Setting: International multi-centre hospital-based study.

Participants: Eligible adults consented and randomised in the TICH-2 trial who were also able to undergo MRI scanning. To address the primary hypothesis a sample size of n=280 will allow detection of a 10% relative increase in prevalence of diffusion weighted imaging hyperintense lesions in the TXA group with 5% significance, 80% power and 5% imaging data rejection.

Interventions: TICH-2 MRI sub-study participants will undergo MRI scanning using a standardised protocol at day ~5 and day ~90 after randomisation. Clinical assessments, randomisation to TXA or placebo and participant follow-up will be performed as per the TICH-2 trial protocol.

Conclusion: The TICH-2 MRI sub-study will test whether TXA increases the incidence of new DWI-defined ischemic lesions or reduces perihaematomal oedema or final ICH lesion volume in the context of SICH.

For peer review only

STRENGTHS AND LIMITATIONS

- Magnetic resonance imaging (MRI) is incorporated into an international multi-centre randomised controlled trial of tranexamic acid (TXA) in spontaneous intracerebral haemorrhage (SICH). This will be used to objectively assess the potential effects of TXA on cerebral ischemia, which might not be visible using standard clinical imaging such as computed tomography.
- This study also uses MRI to assess the potential beneficial effects of TXA on neurotoxicity and neuroinflammation.
- The inclusion of MRI forces the introduction of additional exclusion criteria, which increases the risk of under-recruitment and subsequent reduction of statistical power.

For peer review only

INTRODUCTION AND RATIONALE

Tranexamic acid (TXA) is an antifibrinolytic drug that binds reversibly to the lysine binding site of plasminogen / plasmin [1], leading to potent inhibition of the interaction of plasmin with fibrin that results in reduced fibrinolysis. In the context of spontaneous intracerebral haemorrhage (SICH) it has been postulated that TXA will result in more rapid cessation of bleeding at the point of vessel rupture and hence limit haematoma expansion, a factor shown to be associated with both mortality and disability [2]. The Tranexamic Acid for Hyperacute Primary Intracerebral Haemorrhage (TICH-2) trial is an international multi-centre randomised controlled trial to test the hypothesis that intravenous TXA reduces death and disability when given within 8 hours of SICH (ISRCTN93732214) [3]. However, there are alternative mechanisms by which TXA could alter the pathophysiology, and hence outcome, of the evolving brain injury that accompanies SICH via its interaction with the plasminogen activation axis.

Firstly, there is potential for TXA to precipitate ischaemic events. Diffusion weighted imaging (DWI) MRI hyperintense lesions (DWIHL), which are presumed to be indicative of acute or subacute ischaemia, are known to co-occur with SICH with a prevalence (8-35%) [4–7] and are associated with a higher risk of dependence or death at 3 months (OR 4.8; 95% CI 1.7–13.9; P=0.004) [8]. The mechanisms underlying the high prevalence of DWIHL in SICH are not known. Endothelial failure is thought to be a key event in the mechanism of small vessel disease and lacunar infarction [9]. A sequential process of endothelial failure, non-occlusive wall-associated microthrombosis, vessel rupture, microbleeding, and finally reactive occlusive thrombosis leading to infarction has been proposed based on animal models [10]. The inhibition of fibrin degradation by TXA could potentiate this process

For peer review only - http://bmjopen.bmj.com/site/about/guidelines.xhtml

by exacerbation of microvascular thrombosis. Support for a possible potentiating effect of TXA on cerebral ischaemia comes from a meta-analysis of predominantly prolonged (>10 days) TXA administration in spontaneous subarachnoid haemorrhage (SAH) which found pooled relative risk for reported cerebral ischaemia of 1.41 (95% CI 1.04-1.91) [11]. However multiple studies of TXA in a variety of other disease settings, including traumatic and spontaneous bleeding (non-SAH) showed no increase in cerebral ischaemia with TXA use [12–15].

Secondly, there are potential beneficial effects of TXA by modulation of the plasminogen activation cascade by reducing neurotoxicity and neuroinflammation [16–18]. A study in surgical patients has shown that TXA attenuates the inflammatory response [19], thought to be mediated by inhibition of the plasminogen activation cascade. Clinical and biological markers of inflammatory response at presentation are predictors of early neurological deterioration in SICH [20]. Perihaematomal oedema (PHO) volume and diffusion properties have been proposed as biomarkers of the inflammatory response around SICH [21], and levels of circulating matrix metalloproteinase-3 are independently associated with PHO volume [22]. PHO increases rapidly during the first 48 hours and peaks towards the end of the second week following SICH [23]. MRI DWI of PHO most commonly shows elevated diffusion [24,25] implying that PHO results from increased permeability of the neurovascular unit or alterations in the extravascular ultrastructural environment rather than ischaemia, although in cases where restricted diffusion is detected in PHO an association with poor clinical outcome has been observed [26].

In the TICH-2 MRI study MRI scans will be acquired in a sub-group of the TICH-2 Trial population on day 5 after randomisation (acceptable range day 2 to day 14,

For peer review only

referred to hereafter as the day 5 scan), and day 90 after randomisation (acceptable range day 83 to day 110, referred to as the day 90 scan). The MRI data will be used to test a primary hypothesis regarding treatment-related differences in DWIHL prevalence. It will also allow secondary analyses of treatment effects on PHO volume and diffusion properties, and SICH-related tissue damage at day 90.

Primary hypothesis

Prevalence of remote DWIHL on the day 5 MRI scan will be greater in the TXA group compared to controls.

Secondary hypotheses

1) PHO volume and perihaematomal diffusion restriction on day 5 MRI scan will be reduced in the TXA group compared to controls.

2) SICH-related tissue damage defined on the day 90 MRI scan will be reduced in the TXA group compared to controls, controlling for initial haematoma volume.

We will test whether imaging markers of co-existing small vessel disease (cerebral microbleeds, CMB, and white matter hyperintensities of presumed vascular origin, WMH) and imaging markers of cerebral amyloid angiopathy (CAA; strictly lobar CMB, cortical superficial siderosis) are associated with the presence of DWIHL in TXA treated patients.

For peer review only

DESIGN AND METHODS

Patient population

The recruitment process is summarised in Figure 1. Patients recruited to the main TICH-2 trial according to the TICH-2 trial inclusion and exclusion [3] criteria at centres participating in the TICH-2 MRI study will be invited to participate in the TICH-2 MRI study provided they meet the additional following inclusion / exclusion criteria:

- Additional TICH-2 MRI study inclusion criteria:
    1. participant or delegate freely gives informed consent to participate in the TICH-2 MRI study, or participants within the TICH-2 Trial who have an MRI scan performed for clinical purposes within the MRI substudy time windows using a protocol consistent with the MRI substudy (TICH-2 trial consent includes submission of relevant clinical data which includes imaging data).
    2. Able to undergo MRI scanning.
- Additional TICH-2 MRI study exclusion criteria:
    1. Contraindication to MRI scan (e.g., non-MRI compatible implant, intra-ocular / intracranial metallic device or fragment, claustrophobia, etc.).
    2. Clinical instability (for example cardiorespiratory or neurological instability) such that MRI scan would introduce additional clinical risk.

Randomisation

Participants are randomised into the main TICH-2 study as described previously [3]. Recruitment into the TICH-2 MRI study can occur at any time between the point of initial recruitment to the main TICH-2 study (pre-randomisation) up to day 7 after randomisation. Blinding to treatment allocation is maintained throughout the course of the TICH-2 MRI study.

For peer review only

Intervention

The TICH-2 trial intervention has been described previously [3]. In brief, participants are randomised (1:1) to intravenous tranexamic acid (1g in 100ml IV bolus loading dose followed by 1g in 250ml infusion over 8 hours) or placebo (0.9 % saline using identical administration regime).

	**DWI**	**T2-FLAIR**	**T2* GRE 2D**	**SWI 3D (see note 1)**	**3D T1-Volume**	**T2**	**2D T1 (see note 2)**
**Orientation**	Axial	Axial	Axial	Use standard manufacturer specific SWI protocol from manufacturer's protocol tree (*i.e.,* SWAN (GE); VEN_BOLD (Philips); SWI (Siemens))	Sagittal	Axial	Axial
**Plane**	2D - EPI	2D	2D		3D	2D	2D
**TE** (ms)	Minimum	125-140	20-30 (15-40)		Minimum	85-100	10-14
**TR** (ms)	Minimum	11000	300-1000		Minimum	3000-5600	600-650
**TI** (ms)	NA	2800	NA		450-1000 (or no inversion on old GE FSPGR)	NA	NA
**Slice Thickness** (mm)	⩽ 4.0	⩽ 4.0	3 (3-5)		1mm isotropic voxels (1.25mm)	⩽ 4.0	⩽ 4.0
**Slice Gap** (mm)	⩽ 0.4	⩽ 0.4	⩽ 0.3 (0-1)		NA	⩽ 0.4	⩽ 0.4
**Acquisition Matrix (RFOV acceptable)**	96-128	180-256 (180-512)	180-256		128-256	180-256 (180-512)	180-256
**FOV** (mm)	230-240	230-240	230-240		224-256	230-240	230-240
**Flip Angle** (excitation)	90°	90°	15-60°		8-15°	90°	90°
**Flip Angle** (refocusing)	180°	180°	NA		NA	180°	180°
**b-value**	1000	NA	NA		NA	NA	NA
**Number of slices**	32 - 42	32 - 42	32 - 42		180-192 (128-256)	32 - 42	32 - 42

Note 1 – If available
Note 2 – Only required if 3D T1-volume acquisition is degraded by patient motion

Table 1 - MRI acquisition parameters. Preferred parameters are listed, with acceptable range of values given in brackets for sites where the preferred parameters cannot be achieved for technical reasons.

MRI scanning is performed on day 5 (acceptable range day 2 to day 14) and day 90 (acceptable range day 83 to day 110) post-randomisation. The MRI acquisition protocol (Table 1) complies with the essential imaging sequences specified by the Standards for Reporting Vascular Neuroimaging (STRIVE) standards [9], and includes 2D axial, T2-weighted images, T2*-weighted images, T2-weighted Fluid-attenuated inversion recovery (FLAIR) images, DWI, and a 3D T1-weighted volume

For peer review only

acquisition. Axial 2D T1-weighted images are to be included if the 3D T1-weighted volume is significantly motion degraded, and manufacturer optimised susceptibility weighted imaging (SWI) is to be included if available.

Outcomes

Primary outcome

- Prevalence of remote DWIHL on the day 5 MRI scan

Secondary outcomes

- PHO volume and perihaematomal diffusion restriction on the day 5 MRI scan
- SICH-related tissue damage (the combined volume of the residual haematoma cavity and surrounding FLAIR hyperintensity) determined on the day 90 MRI scan.

Methodology for extraction of the imaging outcome measures is summarised in Figure 2. DWI scans will be analysed for presence, number and distribution of DWIHL. A semi-automated method for quantification of DWIHL will be developed and validated using a subset of patients. It will be subsequently employed to identify candidate lesions based on shape and intensity features. Candidate lesions will then be accepted or rejected by at least two expert readers independently. Only DWIHL that are confirmed of low diffusion on the derived apparent diffusion coefficient maps, and spatially remote from the index ICH (<20mm) will be included as previously [6].

For peer review only

A fully automated segmentation method [27] will exploit the T2, T2* and FLAIR images to segment the haematoma and PHO on the day 5 MRI scan (limited for secondary hypothesis 1 to scans performed up to and including day 7), from which volume and quantification of the diffusion properties of PHO can be derived (Figure 3). FLAIR images will be used to determine the volume of final haematoma cavity and surrounding hyperintensity on the day 90 scan using semi-automated segmentation supervised by experienced image analysts. T1-weighted images will be utilised to determine brain parenchymal volume on the day 90 scan.

WMH will be evaluated on FLAIR and T2-weighted images using an established 4 point scale [28] and automated WMH segmentation will be used for WMH volume calculation and creation of a distribution map of WMH. CMB number and distribution will be rated using Microbleed Anatomical Rating Scale (MARS) and used for classification of ICH patients into probable CAA and non-CAA groups [29].

Sample size estimates

Primary hypothesis: Based on previous studies finding prevalence of DWIHL of 20% in SICH, we performed calculations for sample size to allow us to detect different percentage increases in DWIHL prevalence in the TXA group above a 20% baseline, using the sample size formula for prevalence:

$$n = \frac{(z^2_{1-\alpha/2}) \cdot p(1-p)}{m^2}, \quad (1)$$

where *z* = confidence interval, *p* = prevalence, and *m* = margin of error (standard is 5%). Assuming a small relative increase in prevalence of DWIHL by 10% in the TXA group, with 5% significance and 80% power, a sample size of 264 is required. Allowing 5% imaging data rejection (e.g. due to excessive patient motion), a sample size of 280 is required.

For peer review only - http://bmjopen.bmj.com/site/about/guidelines.xhtml

Secondary hypotheses: Our pilot data from the TICH-1 study [30] showed that for every 1ml of SICH volume, the mean relative PHO volume increased between the Day 0 and Day 2 CT scan by 0.29ml (standard deviation, s.d. 0.34) in TXA-treated patients and by 0.41ml (s.d. 0.27ml) in the placebo group. Assuming a similar effect size is present on the day 5 MRI scan using the sample size proposed for the main hypothesis (n=280) we will be able to detect a group difference in the increase in relative PHO with power of 0.89 and α=0.05 (independent samples t-test, 2-tailed).

Statistical analyses

To analyse the primary hypothesis, presence of DWIHL post-SICH will be compared between treatment groups using a binary logistic regression with adjustment for a selection of baseline covariates; age, time from onset to randomisation, mean systolic blood pressure, known history of antiplatelet treatment and baseline haematoma volume.

To test the secondary hypotheses, a group comparison test of PHO volume, PHO diffusion metrics and day 90 combined haematoma cavity and surrounding FLAIR hyperintensity volume between participants in the TXA and control treatment groups will be performed. Other proposed analyses will include univariate analyses of association between presence of DWIHL and imaging markers of small vessel disease (CMB, WMH) and likely diagnostic classification for CAA (based on CMB distribution and presence of superficial siderosis); and exploratory analyses of associations between imaging outcomes and clinical outcomes, with adjustment for haematoma expansion. All regression analyses will be adjusted for the covariates listed previously.

For peer review only

Study organization and funding

The TICH-2 MRI study is funded by a grant from British Heart Foundation (grant reference PG/14/96/31262). The TICH-2 trial is funded by a grant from the UK National Institute for Health Research Health Technology Assessment programme (project code 11_129_109). The TICH-2 trial (ISRCTN50867461) obtained ethical approval from Nottingham-2 Research Ethics Committee on 19/11/12, and an amendment to allow the TICH-2 MRI substudy was approved on 21/04/2015 (amendment number SA02/15). The University of Nottingham acts as the trial sponsor. The trial steering committee for the TICH-2 trial oversees the TICH-2 MRI substudy. Data monitoring for the TICH-2 trial is performed by an independent data monitoring committee [3]. Recruitment into the MRI substudy started in July 2015 and is due to end in September 2017.

DISCUSSION

TXA may have biological effects in the brain tissue that could alter the outcome in SICH patients, independent to the postulated effects on haematoma expansion. The main theoretical concern is that the haemostatic effects of TXA might increase the risk of cerebral ischaemia. Understanding these effects is vital, particularly if the results of ongoing trials [31] show an effect of TXA on outcome that is not (or only partially) explained by an effect of TXA on haematoma expansion. This study allows us to quantify treatment related brain changes (particularly prevalence of ischaemic lesions, PHO volume and diffusion characteristics, and late post-SICH tissue damage) in SICH patients in the context of a multicentre randomised controlled trial. If the results of the ongoing trials support the use of TXA, then the opportunity to study the potential therapeutic effects of TXA in acute cerebrovascular brain injury in

For peer review only

a controlled trial is unlikely to occur again. By including imaging markers of small vessel disease and CAA in the analysis of DWIHL burden, we will be able to test whether there is a subgroup of patients at risk of DWIHL following TXA treatment for SICH, which will inform future studies of TXA in SICH and could ultimately influence clinical practice. If the study shows a beneficial effect of TXA on PHO, then this study may trigger studies of TXA in other acute brain pathologies associated with oedema and inflammation.

SUMMARY AND CONCLUSION

TXA is a widely available and inexpensive antifibrinolytic drug, which has the potential of reducing neurotoxicity and neuroinflammation in SICH. However, it is also possible that it may potentiate the risk of ischaemic events. The TICH-2 MRI substudy will assess the effectiveness of TXA in reducing inflammatory response, while also determining whether there is an increased risk of ischaemic events following its administration. This will inform future studies of TXA and, if found to be effective and safe, have an important impact in clinical practice.

AUTHORS' CONTRIBUTION

RAD, KF, PSM, IR, DW, RAS, TE, PMB and NS designed the study and were co-applicants for funding. SP designed and will perform the image analyses. KF designed and will perform the statistical analyses. RAD, SP, ZKL, KF and NS drafted the manuscript. All authors reviewed and commented on the manuscript.

For peer review only

COMPETING INTERESTS

The authors have no conflicts of interest to disclose with respect to the research, authorship and publication of this article.

For peer review only

REFERENCES

1 McCormack PL. Tranexamic Acid: A review of its use in the treatment of hyperfibrinolysis. *Drugs* 2012;**72**:585–617.

2 Davis SM, Broderick J, Hennerici M, *et al.* Hematoma growth is a determinant of mortality and poor outcome after intracerebral hemorrhage. *Neurology* 2006;**66**:1175–81.

3 Sprigg N, Robson K, Bath PM, *et al.* Intravenous tranexamic acid for hyperacute primary intracerebral hemorrhage: Protocol for a randomized, placebo-controlled trial. *Int J stroke* 2016;**11**:717–23.

4 Prabhakaran S, Gupta R, Ouyang B, *et al.* Acute brain infarcts after spontaneous intracerebral hemorrhage: A diffusion-weighted imaging study. *Stroke* 2010;**41**:89–94.

5 Menon RS, Burgess RE, Wing JJ, *et al.* Predictors of highly prevalent brain ischemia in intracerebral hemorrhage. *Ann Neurol* 2012;**71**:199–205.

6 Gregoire SM, Charidimou A, Gadapa N, *et al.* Acute ischaemic brain lesions in intracerebral haemorrhage: Multicentre cross-sectional magnetic resonance imaging study. *Brain* 2011;**134**:2376–86.

7 Kang DW, Han MK, Kim HJ, *et al.* New ischemic lesions coexisting with acute intracerebral hemorrhage. *Neurology* 2012;**79**:848–55.

8 Garg RK, Liebling SM, Maas MB, *et al.* Blood pressure reduction, decreased diffusion on MRI, and outcomes after intracerebral hemorrhage. *Stroke* 2012;**43**:67–71.

9 Wardlaw JM, Smith EE, Biessels GJ, *et al.* Neuroimaging standards for research into small vessel disease and its contribution to ageing and neurodegeneration. *Lancet Neurol* 2013;**12**:822–38.

10 Schreiber S, Bueche CZ, Garz C, *et al.* Blood brain barrier breakdown as the

For peer review only

starting point of cerebral small vessel disease? - New insights from a rat model. *Exp Transl Stroke Med* 2013;**5**:4.

11 Baharoglu MI, Germans MR, Rinkel GJE, *et al.* Antifibrinolytic therapy for aneurysmal subarachnoid haemorrhage. *Cochrane database Syst Rev* 2013.

12 Ross J, Al-Shahi Salman R. The frequency of thrombotic events among adults given antifibrinolytic drugs for spontaneous bleeding: Systematic review and meta-analysis of observational studies and randomized trials. *Curr Drug Saf* 2012;**7**:44–54.

13 Perel P, Al-Shahi Salman R, Kawahara T, *et al.* CRASH-2 (Clinical Randomisation of an Antifibrinolytic in Significant Haemorrhage) intracranial bleeding study: the effect of tranexamic acid in traumatic brain injury--a nested randomised, placebo-controlled trial. *Health Technol Assess* 2012;**16**.

14 CRASH-2 Collaborators. Effect of tranexamic acid in traumatic brain injury: a nested randomised, placebo controlled trial (CRASH-2 Intracranial Bleeding Study). *BMJ* 2011;**343**:d3795.

15 Roberts I, Shakur H, Ker K, *et al.* Antifibrinolytic drugs for acute traumatic injury. *Cochrane database Syst Rev* 2011;**12**:CD004896.

16 Lo EH, Wang X, Cuzner ML. Extracellular proteolysis in brain injury and inflammation: Role for plasminogen activators and matrix metalloproteinases. *J Neurosci Res* 2002;**69**:1–9.

17 Chen ZL, Strickland S. Neuronal death in the hippocampus is promoted by plasmin-catalyzed degradation of laminin. *Cell* 1997;**91**:917–25.

18 Endo A, Nagai N, Urano T, *et al.* Proteolysis of neuronal cell adhesion molecule by the tissue plasminogen activator-plasmin system after kainate injection in the mouse hippocampus. *Neurosci Res* 1999;**33**:1–8.

19 Later AFL, Sitniakowsky LS, van Hilten JA, *et al.* Antifibrinolytics attenuate inflammatory gene expression after cardiac surgery. *J Thorac Cardiovasc Surg*

For peer review only

2013;**145**:1611–1616.e4.

20 Leira R, Dávalos A, Silva Y, *et al.* Early neurologic deterioration in intracerebral hemorrhage: Predictors and associated factors. *Neurology* 2004;**63**:461–7.

21 Fu Y, Hao J, Zhang N, *et al.* Fingolimod for the treatment of intracerebral hemorrhage: A 2-arm proof-of-concept study. *JAMA Neurol* 2014;**71**:1–10.

22 Li N, Liu Y., Ma L, *et al.* Association of molecular markers with perihematomal edema and clinical outcome in intracerebral hemorrhage. *Stroke* 2013;**44**:658–63.

23 Venkatasubramanian C, Mlynash M, Finley-Caulfield A, *et al.* Natural history of perihematomal edema following intracerebral hemorrhage measured by serial magnetic resonance imaging. *Stroke* 2011;**42**:73–80.

24 Butcher KS, Baird T, MacGregor L, *et al.* Perihematomal edema in primary intracerebral hemorrhage is plasma derived. *Stroke* 2004;**35**:1879–85.

25 Carhuapoma JR, Barker PB, Hanley DF, *et al.* Human brain hemorrhage: Quantification of perihematoma edema by use of diffusion-weighted MR imaging. *Am J Neuroradiol* 2002;**23**:1322–6.

26 Kidwell CS, Saver JL, Mattiello J, *et al.* Diffusion-perfusion MR evaluation of perihematomal injury in hyperacute intracerebral hemorrhage. *Neurology* 2001;**57**:1611–7.

27 Pszczolkowski S, Gallagher RG, Law ZK, *et al.* Automatic Quantification of Haematoma and Surrounding Oedema in MRI of Acute Spontaneous Intracerebral Haemorrhage: Preliminary Results for the TICH-2 MRI Sub-study. In: *Proc. ISMRM*. 2017.

28 Wahlund LO, Barkhof F, Fazekas F, *et al.* A new rating scale for age-related white matter changes applicable to MRI and CT. *Stroke* 2001;**6**:1318–23.

29 Gregoire SM, Chaudhary UJ, Brown MM, *et al.* The Microbleed Anatomical Rating Scale (MARS): Reliability of a tool to map brain microbleeds. *Neurology*

For peer review only

2009;**73**:1759–66.

30 Sprigg N, Renton CJ, Dineen RA, *et al.* Tranexamic acid for spontaneous intracerebral hemorrhage: A randomized controlled pilot trial (ISRCTN50867461). *J Stroke Cerebrovasc Dis* 2014;**23**:1312–8.

31 Law ZK, Meretoja A, Engelter ST, *et al.* Treatment of intracerebral haemorrhage with tranexamic acid - A review of current evidence and ongoing trials. *Eur Stroke J* 2017;**2**:13–22.

For peer review only

Figure 1 – TICH-2 MRI substudy flowchart. Details of participant flow for the TICH-2 trial, including the schedule for randomisation, treatment, cranial CT, clinical assessments and non-MRI follow-up assessments have been described in Sprigg et al [3].

Figure 2 – Summary of image processing and outputs in the TICH-2 MRI substudy.

Figure 3 - Measurement of perihaematomal oedema (PHO) volume and apparent diffusion coefficient (ADC). (a) Axial FLAIR image in native T1 space showing the PHO delineation from which the PHO volume can be derived. (b) ADC map in native T1 space. PHO ADC values are calculated from the voxels within the PHO delineation in the ADC map.

For peer review only

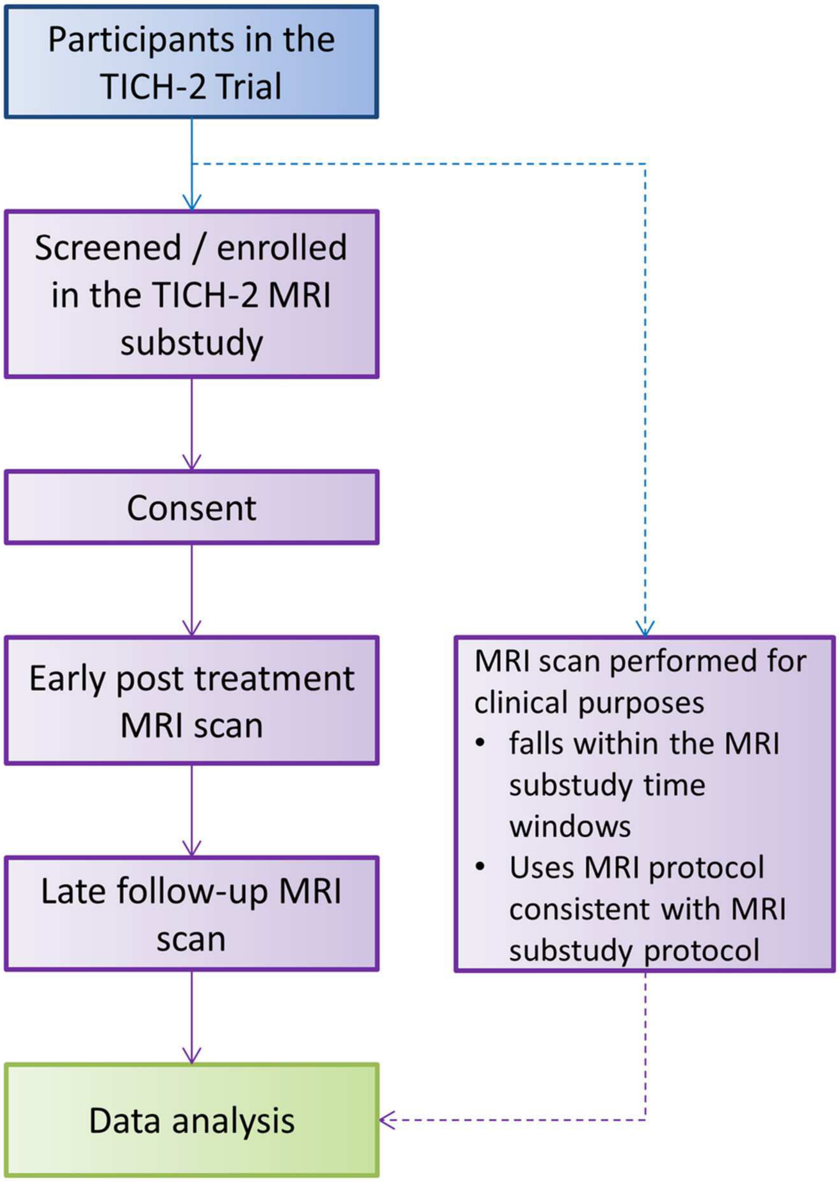

TICH-2 MRI substudy flowchart. Details of participant flow for the TICH-2 trial, including the schedule for randomisation, treatment, cranial CT, clinical assessments and non-MRI follow-up assessments have been described in Sprigg et al [3].

42x59mm (600 x 600 DPI)

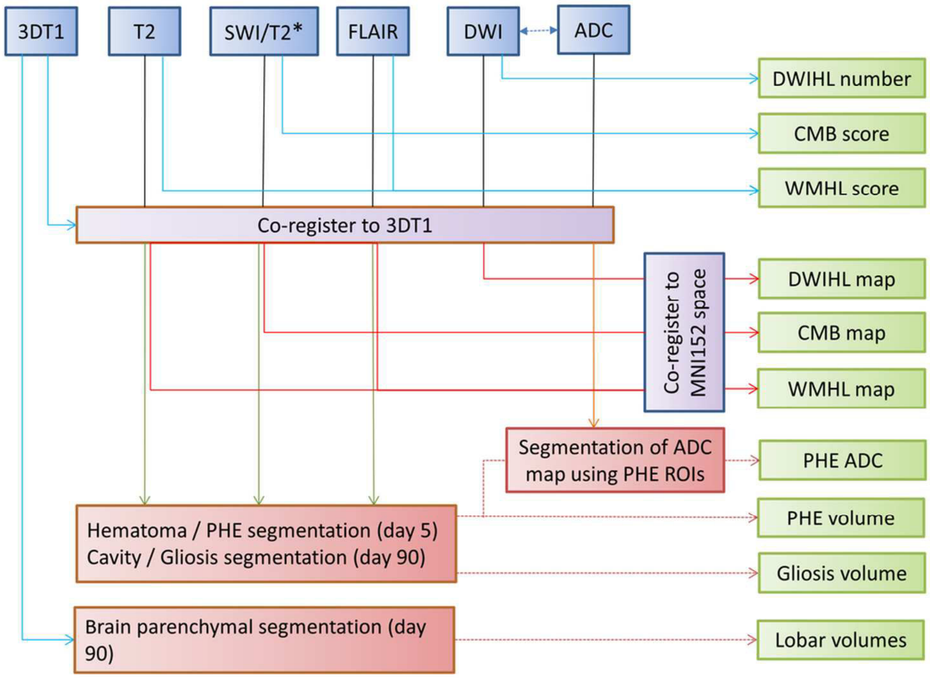

Summary of image processing and outputs in the TICH-2 MRI substudy.

47x35mm (600 x 600 DPI)

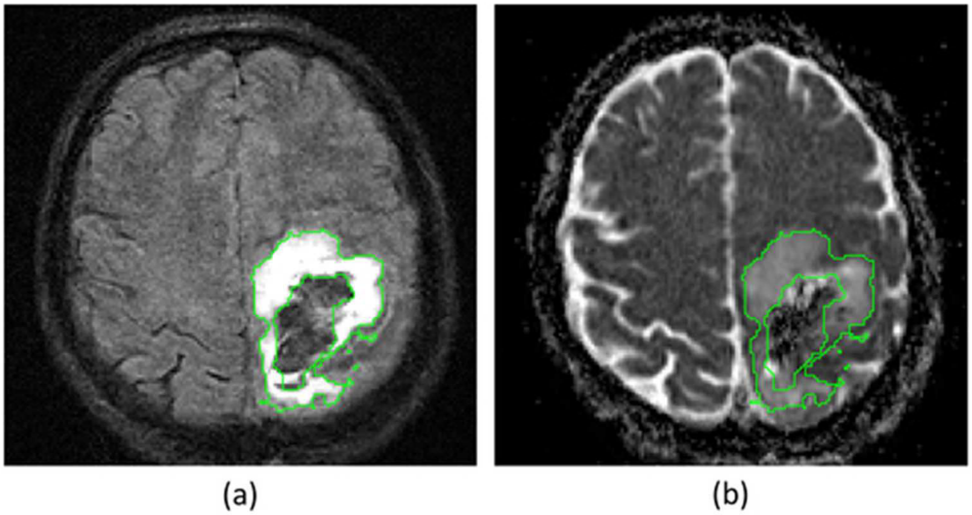

Measurement of perihaematomal oedema (PHO) volume and apparent diffusion coefficient (ADC). (a) Axial FLAIR image in native T1 space showing the PHO delineation from which the PHO volume can be derived. (b) ADC map in native T1 space. PHO ADC values are calculated from the voxels within the PHO delineation in the ADC map.

24x13mm (600 x 600 DPI)

eview only

BMJ Open

Does tranexamic acid lead to changes in MRI-measures of brain tissue health in patients with spontaneous intracerebral haemorrhage? Protocol for a MRI sub-study nested within the double-blind randomised controlled TICH-2 trial

Journal:	*BMJ Open*
Manuscript ID	bmjopen-2017-019930.R1
Article Type:	Protocol
Date Submitted by the Author:	28-Nov-2017

Complete List of Authors:	Dineen, Rob; University of Nottingham School of Medicine, Division of Clinical Neuroscience<br>Pszczolkowski, Stefan; University of Nottingham School of Medicine, Division of Clinical Neuroscience<br>Flaherty, Katie; University of Nottingham School of Medicine, Division of Clinical Neuroscience<br>Law, Zhe; University of Nottingham School of Medicine, Division of Clinical Neuroscience<br>Morgan, Paul; University of Nottingham School of Medicine, Division of Clinical Neuroscience<br>Roberts, Ian; London School of Hygiene and Tropical Medicine, Epidemiology and Population Health<br>Werring, David; University College London, Stroke Research Centre<br>Salman, Rustam; University of Edinburgh, Centre for Clinical Brain Sciences<br>England, Tim; University of Nottingham School of Medicine, Vascular Medicine, Division of Medical Sciences & GEM<br>Bath, Philip; University of Nottingham School of Medicine, Division of Clinical Neuroscience<br>Sprigg, Nicola; University of Nottingham School of Medicine, Division of Clinical Neuroscience

**Primary Subject Heading**:	Radiology and imaging
Secondary Subject Heading:	Radiology and imaging, Neurology
Keywords:	hyperacute primary intracerebral haemorrhage, tranexamic acid, Magnetic resonance imaging < RADIOLOGY & IMAGING, diffusion weighted imaging, perihaematomal oedema

SCHOLARONE™ Manuscripts

Title:

Does tranexamic acid lead to changes in MRI-measures of brain tissue health in patients with spontaneous intracerebral haemorrhage? An MRI sub-study nested within the double-blind randomised controlled TICH-2 trial.

Authors:

Rob A. Dineen[1,2,3], Stefan Pszczolkowski[1,4], Katie Flaherty[4], Zhe K. Law[4,5], Paul S. Morgan[1,2,6], Ian Roberts[7], David Werring[8], Rustam Al-Shahi Salman[9], Tim England[10], Philip M. Bath[4], Nikola Sprigg[4]

[1]*Radiological Sciences, Division of Clinical Neuroscience, University of Nottingham, Nottingham, UK*

[2]*Sir Peter Mansfield Imaging Centre, University of Nottingham, Nottingham, UK*

[3]*NIHR Nottingham Biomedical Research Centre, Nottingham, UK*

[4]*Stroke Trials Unit, Division of Clinical Neuroscience, University of Nottingham, Nottingham, UK*

[5]*National University of Malaysia, Kuala Lumpur, Malaysia*

[6]*Medical Physics and Clinical Engineering, Nottingham University Hospitals NHS Trust, Nottingham, UK*

[7]*London School of Hygiene & Tropical Medicine, London, UK*

[8]*Stroke Research Centre, University College London, London, UK*

[9]*Centre for Clinical Brain Sciences, University of Edinburgh, Edinburgh, UK*

[10]*Vascular Medicine, Division of Medical Sciences & GEM, University of Nottingham, Nottingham, UK*

Correspondence: Dr. Rob Dineen, Division of Clinical Neuroscience, University of Nottingham, Queen's Medical Centre, Derby Road, Nottingham NG7 2UH, UK.

For peer review only

Email rob.dineen@nottingham.ac.uk

Key words:

hyperacute primary intracerebral haemorrhage, tranexamic acid, magnetic resonance imaging, diffusion weighted imaging, perihaematomal oedema

Word count

2416 words for main text

For peer review only

ABSTRACT

Objectives: To test whether administration of the antifibrinolytic drug tranexamic acid (TXA) in patients with spontaneous intracerebral haemorrhage (SICH) leads to increased prevalence of diffusion-weighted MRI defined hyperintense ischaemic lesions (primary hypothesis) or reduced perihaematomal oedema volume, perihaematomal diffusion restriction and residual MRI-defined SICH-related tissue damage (secondary hypotheses).

Design: MRI sub-study nested within the double-blind randomised controlled TICH-2 trial (ISRCTN93732214).

Setting: International multi-centre hospital-based study.

Participants: Eligible adults consented and randomised in the TICH-2 trial who were also able to undergo MRI scanning. To address the primary hypothesis a sample size of n=280 will allow detection of a 10% relative increase in prevalence of diffusion weighted imaging hyperintense lesions in the TXA group with 5% significance, 80% power and 5% imaging data rejection.

Interventions: TICH-2 MRI sub-study participants will undergo MRI scanning using a standardised protocol at day ~5 and day ~90 after randomisation. Clinical assessments, randomisation to TXA or placebo and participant follow-up will be performed as per the TICH-2 trial protocol.

Conclusion: The TICH-2 MRI sub-study will test whether TXA increases the incidence of new DWI-defined ischemic lesions or reduces perihaematomal oedema or final ICH lesion volume in the context of SICH.

For peer review only

STRENGTHS AND LIMITATIONS

- Magnetic resonance imaging (MRI) is incorporated into an international multi-centre randomised controlled trial of tranexamic acid (TXA) in spontaneous intracerebral haemorrhage (SICH). This will be used to objectively assess the potential effects of TXA on cerebral ischemia, which might not be visible using standard clinical imaging such as computed tomography.
- This study also uses MRI to assess any potential beneficial effects of TXA on neurotoxicity and neuroinflammation.
- The inclusion of MRI necessitates the introduction of additional exclusion criteria, which increases the risk of under-recruitment and subsequent reduction of statistical power.
- A limitation of the recruitment process to the TICH-2 MRI sub-study is that recruitment took place after-randomisation. As a result survivor bias may be a potential confounder, which we address by conducting regression analyses adjusting for baseline variables.

For peer review only

INTRODUCTION AND RATIONALE

Tranexamic acid (TXA) is an antifibrinolytic drug that binds reversibly to the lysine binding site of plasminogen / plasmin [1], leading to potent inhibition of the interaction of plasmin with fibrin that results in reduced fibrinolysis. In the context of spontaneous intracerebral haemorrhage (SICH) it has been postulated that TXA will result in more rapid cessation of bleeding at the point of vessel rupture and hence limit haematoma expansion, a factor shown to be associated with both mortality and disability [2]. The Tranexamic Acid for Hyperacute Primary Intracerebral Haemorrhage (TICH-2) trial is an international multi-centre randomised controlled trial to test the hypothesis that intravenous TXA reduces death and disability when given within 8 hours of SICH (ISRCTN93732214) [3]. However, there are alternative mechanisms in addition to haemostatic by which TXA, could alter the pathophysiology, and hence outcome, of the evolving brain injury that accompanies SICH via its interaction with the plasminogen activation axis.

Firstly, patients with SICH are at risk of co-occurring cerebral ischaemic events, and TXA could theoretically potentiate this risk by the inhibition of fibrin degradation. Diffusion weighted imaging (DWI) MRI hyperintense lesions (DWIHL), which are thought to be indicative of acute or subacute ischaemia, co-occur with SICH with a prevalence (8-35%) [4–7] and are associated with a higher risk of dependence or death at 3 months (OR 4.8; 95% CI 1.7–13.9; *P*=0.004) [8]. The mechanisms underlying the high prevalence of DWIHL in SICH are not known. Endothelial failure is thought to be a key event in the mechanism of small vessel disease and lacunar infarction [9]. A sequential process of endothelial failure, non-occlusive wall-associated microthrombosis, vessel rupture, microbleeding, and finally reactive occlusive thrombosis leading to infarction has been proposed based on animal

For peer review only

models [10]. The inhibition of fibrin degradation by TXA could potentiate this process by exacerbation of microvascular thrombosis. Support for a possible potentiating effect of TXA on cerebral ischaemia comes from a meta-analysis of predominantly prolonged (>10 days) TXA administration in spontaneous subarachnoid haemorrhage (SAH) which found pooled relative risk for reported cerebral ischaemia of 1.41 (95% CI 1.04-1.91) [11]. However multiple studies of TXA in a variety of other disease settings, including traumatic and spontaneous bleeding (non-SAH) showed no increase in cerebral ischaemia with TXA use [12–15].

Secondly, there are potentially beneficial effects of TXA by modulation of the plasminogen activation cascade by reducing neurotoxicity and neuroinflammation [16–18]. A study in surgical patients has shown that TXA attenuates the inflammatory response [19], thought to be mediated by inhibition of the plasminogen activation cascade. Clinical and biological markers of inflammatory response at presentation are predictors of early neurological deterioration in SICH [20]. Perihaematomal oedema (PHO) volume and diffusion properties have been proposed as biomarkers of the inflammatory response around SICH [21], and levels of circulating matrix metalloproteinase-3 are independently associated with PHO volume [22]. PHO increases rapidly during the first 48 hours and peaks towards the end of the second week following SICH [23]. MRI DWI of PHO most commonly shows elevated diffusion [24,25] implying that PHO results from increased permeability of the neurovascular unit or alterations in the extravascular ultrastructural environment rather than ischaemia, although in cases where restricted diffusion is detected in PHO an association with poor clinical outcome has been observed [26].

For peer review only

In the TICH-2 MRI sub-study MRI scans will be acquired in a sub-group of the TICH-2 Trial population on day 5 after randomisation (acceptable range day 2 to day 14, referred to hereafter as the day 5 scan), and day 90 after randomisation (acceptable range day 83 to day 110, referred to as the day 90 scan). The MRI data will be used to test a primary hypothesis regarding treatment-related differences in DWIHL prevalence. It will also allow secondary analyses of treatment effects on PHO volume and diffusion properties, and SICH-related tissue damage at day 90.

Primary hypothesis

Prevalence of remote DWI hyperintense lesions on the day 5 MRI scan will be greater in the TXA group compared to controls.

Secondary hypotheses

1) Perihaematomal oedema volume and perihaematomal diffusion restriction on day 5 MRI scan will be reduced in the TXA group compared to controls.
2) SICH-related tissue damage defined on the day 90 MRI scan will be reduced in the TXA group compared to controls, controlling for initial haematoma volume.

We will test whether imaging markers of co-existing small vessel disease (cerebral microbleeds, CMB, and white matter hyperintensities of presumed vascular origin, WMH) and imaging markers of cerebral amyloid angiopathy (CAA; strictly lobar CMB, cortical superficial siderosis) are associated with the presence of DWIHL in TXA treated patients.

For peer review only

DESIGN AND METHODS

Patient population

The recruitment process is summarised in Figure 1. Patients recruited to the main TICH-2 trial according to the TICH-2 trial inclusion and exclusion criteria [3] (see supplementary information) at centres participating in the TICH-2 MRI sub-study will be invited to participate in the TICH-2 MRI sub-study provided they meet the additional following inclusion / exclusion criteria:

- Additional TICH-2 MRI study inclusion criteria:
  1. Participant or delegate freely gives informed consent to participate in the TICH-2 MRI sub-study, or participants within the TICH-2 Trial who have an MRI scan performed for clinical purposes within the MRI sub-study time windows using a protocol consistent with the MRI sub-study (TICH-2 trial consent includes submission of relevant clinical data which includes imaging data).
  2. Able to undergo MRI scanning.
- Additional TICH-2 MRI sub-study exclusion criteria:
  1. Contraindication to MRI scan (e.g., non-MRI compatible implant, intra-ocular / intracranial metallic device or fragment, claustrophobia, etc.).
  2. Clinical instability (for example cardiorespiratory or neurological instability) such that MRI scan would introduce additional clinical risk.

Randomisation

Participants are randomised into the main TICH-2 study as described previously [3]. Recruitment into the TICH-2 MRI sub-study can occur at any time between the point of initial recruitment to the main TICH-2 study (pre-randomisation) up to day 7 after randomisation. Blinding to treatment allocation is maintained throughout the course of both the TICH-2 main and MRI sub-study.

For peer review only

Intervention

The TICH-2 trial intervention has been described previously [3]. In brief, participants are randomised (1:1) to intravenous tranexamic acid (1g in 100ml IV bolus loading dose followed by 1g in 250ml infusion over 8 hours) or placebo (0.9 % saline using identical administration regime).

	DWI	T2-FLAIR	T2* GRE 2D	SWI 3D (see note 1)	3D T1-Volume	T2	2D T1 (see note 2)
**Orientation**	Axial	Axial	Axial	Use standard manufacturer specific SWI protocol from manufacturer's protocol tree (*i.e.*, SWAN (GE); VEN_BOLD (Philips); SWI (Siemens))	Sagittal	Axial	Axial
**Plane**	2D - EPI	2D	2D		3D	2D	2D
**TE** (ms)	Minimum	125-140	20-30 (15-40)		Minimum	85-100	10-14
**TR** (ms)	Minimum	11000	300-1000		Minimum	3000-5600	600-650
**TI** (ms)	NA	2800	NA		450-1000 (or no inversion on old GE FSPGR)	NA	NA
**Slice Thickness** (mm)	≤ 4.0	≤ 4.0	3 (3-5)		1mm isotropic voxels (1.25mm)	≤ 4.0	≤ 4.0
**Slice Gap** (mm)	≤ 0.4	≤ 0.4	≤ 0.3 (0-1)		NA	≤ 0.4	≤ 0.4
**Acquisition Matrix (RFOV acceptable)**	96-128	180-256 (180-512)	180-256		128-256	180-256 (180-512)	180-256
**FOV** (mm)	230-240	230-240	230-240		224-256	230-240	230-240
**Flip Angle** (excitation)	90°	90°	15-60°		8-15°	90°	90°
**Flip Angle** (refocusing)	180°	180°	NA		NA	180°	180°
**b-value**	1000	NA	NA		NA	NA	NA
**Number of slices**	32 - 42	32 - 42	32 - 42		180-192 (128-256)	32 - 42	32 - 42

Note 1 – If available
Note 2 – Only required if 3D T1-volume acquisition is degraded by patient motion

Table 1 - MRI acquisition parameters. Preferred parameters are listed, with acceptable range of values given in brackets for sites where the preferred parameters cannot be achieved for technical reasons.

For peer review only

MRI scanning is performed on day 5 (acceptable range day 2 to day 14) and day 90 (acceptable range day 83 to day 110) post-randomisation. The MRI acquisition protocol (Table 1) complies with the essential imaging sequences specified by the Standards for Reporting Vascular Neuroimaging (STRIVE) standards [9], and includes 2D axial, T2-weighted images, T2*-weighted images, T2-weighted Fluid-attenuated inversion recovery (FLAIR) images, DWI, and a 3D T1-weighted volume

acquisition. Axial 2D T1-weighted images are to be included if the 3D T1-weighted volume is significantly motion degraded, and manufacturer optimised susceptibility weighted imaging (SWI) is to be included if available.

Outcomes

Primary outcome

- Prevalence of remote DWIHL on the day 5 MRI scan

Secondary outcomes

- Perihaematomal oedema volume and perihaematomal diffusion restriction on the day 5 MRI scan
- SICH-related tissue damage (the combined volume of the residual haematoma cavity and surrounding FLAIR hyperintensity) determined on the day 90 MRI scan.

Methodology for extraction of the imaging outcome measures is summarised in Figure 2. DWI scans will be analysed for presence, number and distribution of DWIHL. A semi-automated method for quantification of DWIHL will be developed and validated using a subset of patients. It will be subsequently employed to identify candidate lesions based on shape and intensity features. Candidate lesions will then be accepted or rejected by at least two expert readers independently. Only DWIHL that are confirmed of low diffusion on the derived apparent diffusion coefficient maps, and spatially remote from the index ICH (<20mm) will be included as previously [6].

For peer review only

A fully automated segmentation method [27] will exploit the T2, T2* and FLAIR images to segment the haematoma and PHO on the day 5 MRI scan (limited for secondary hypothesis 1 to scans performed up to and including day 7), from which volume and quantification of the diffusion properties of PHO can be derived (Figure 3). FLAIR images will be used to determine the volume of final haematoma cavity and surrounding hyperintensity on the day 90 scan using semi-automated segmentation supervised by experienced image analysts. T1-weighted images will be utilised to determine brain parenchymal volume on the day 90 scan.

WMH will be evaluated on FLAIR and T2-weighted images using an established 4 point scale [28] and automated WMH segmentation will be used for WMH volume calculation and creation of a distribution map of WMH. CMB number and distribution will be rated using Microbleed Anatomical Rating Scale (MARS) and used for classification of ICH patients into probable CAA and non-CAA groups [29].

Sample size estimates

Primary hypothesis: Based on previous studies finding prevalence of DWIHL of 20% in SICH, we performed calculations for sample size to allow us to detect different percentage increases in DWIHL prevalence in the TXA group above a 20% baseline, using the sample size formula for prevalence:

$$n = \frac{(z^2_{1-\alpha/2}) \cdot p(1-p)}{m^2}, \qquad (1)$$

where z = confidence interval, p = prevalence, and m = margin of error (standard is 5%). Assuming a 10% relative increase in prevalence of DWIHL (i.e. from 20% to 22%) in the TXA group, with 5% significance and 80% power, a sample size of 264 is required. Allowing 5% imaging data rejection (e.g. due to excessive patient motion), a sample size of 280 is required.

For peer review only

Secondary hypotheses: Our pilot data from the TICH-1 study [30] showed that for every 1ml of SICH volume, the mean relative PHO volume increased between the Day 0 and Day 2 CT scan by 0.29ml (standard deviation, s.d. 0.34) in TXA-treated patients and by 0.41ml (s.d. 0.27ml) in the placebo group. Assuming a similar effect size is present on the day 5 MRI scan using the sample size proposed for the main hypothesis (n=280) we will be able to detect a group difference in the increase in relative PHO with power of 0.89 and $\alpha=0.05$ (independent samples t-test, 2-tailed).

Statistical analyses

Group baseline characteristics will be compared between the TICH-2 MRI sub-study and the main TICH-2 trial to examine the extent to which the TICH-2 MRI sub-study participants are representative of the TICH-2 trial populations. To analyse the primary hypothesis, presence of DWIHL post-SICH will be compared between treatment groups using a binary logistic regression with adjustment for a selection of baseline covariates; age, time from onset to randomisation, stroke severity (using National Institutes of Health Stroke Scale), mean systolic blood pressure, known history of antiplatelet treatment and baseline haematoma volume.

To test the secondary hypotheses, a group comparison test of PHO volume, PHO diffusion metrics and day 90 combined haematoma cavity and surrounding FLAIR hyperintensity volume between participants in the TXA and control treatment groups will be performed. Other proposed analyses will include analyses of association between presence of DWIHL and imaging markers of small vessel disease (CMB, WMH) and likely diagnostic classification for CAA (based on CMB distribution and presence of superficial siderosis); and exploratory analyses of associations between

For peer review only

imaging outcomes and clinical outcomes, with adjustment for haematoma expansion. All regression analyses will be adjusted for the covariates listed previously.

Study organization and funding

The TICH-2 MRI sub-study is funded by a grant from British Heart Foundation (grant reference PG/14/96/31262). The TICH-2 trial is funded by a grant from the UK National Institute for Health Research Health Technology Assessment programme (project code 11_129_109). The TICH-2 trial (ISRCTN50867461) obtained ethical approval from Nottingham-2 Research Ethics Committee on 19/11/12, and an amendment to allow the TICH-2 MRI sub-study was approved on 21/04/2015 (amendment number SA02/15). The University of Nottingham acts as the trial sponsor. The trial steering committee for the TICH-2 trial oversees the TICH-2 MRI sub-study. Data monitoring for the TICH-2 trial is performed by an independent data monitoring committee [3]. Recruitment into the MRI sub-study started in July 2015 and is due to end in September 2017.

DISCUSSION

Tranexamic acid may have biological effects in the brain tissue that could alter the outcome in SICH patients, independent to the postulated effects on haematoma expansion. The main theoretical concern is that the haemostatic effects of TXA might increase the risk of cerebral ischaemia. Understanding these effects is vital, particularly if the results of ongoing trials [31] show an effect of TXA on outcome that is not (or only partially) explained by an effect of TXA on haematoma expansion. This study allows us to quantify treatment related brain changes (particularly prevalence of ischaemic lesions, PHO volume and diffusion characteristics, and late post-SICH tissue damage) in SICH patients in the context of a multicentre

For peer review only - http://bmjopen.bmj.com/site/about/guidelines.xhtml

randomised controlled trial. If the results of the ongoing trials support the use of TXA, then the opportunity to study the potential therapeutic effects of TXA in acute cerebrovascular brain injury in a controlled trial is unlikely to occur again. By including imaging markers of small vessel disease and CAA in the analysis of DWIHL burden, we will be able to test whether there is a subgroup of patients at risk of DWIHL following TXA treatment for SICH, which will inform future studies of TXA in SICH and could ultimately influence clinical practice. If the study shows a beneficial effect of TXA on PHO, then this study may trigger studies of TXA in other acute brain pathologies associated with oedema and inflammation.

A limitation of the recruitment process to the TICH-2 MRI sub-study is that recruitment took place after-randomisation. As a result survivor bias could be a confounder as a treatment allocation-related impact on survival of one group over the other could lead to imbalance between the groups entering into the study. Recruitment to the MRI sub-study pre-randomisation was discussed by the investigators during the study design but was considered to be impractical due to the requirement for additional information to be delivered to the participant / relatives and additional consent pre-randomisation which could potentially lead to delays to randomisation for the main TICH-2 trial. To identify a potential survivor bias we will compare group baseline characteristics between the participants in the TICH-2 MRI sub-study and the main TICH-2 trial. To attempt to control for possible imbalances we conduct the regression analyses adjusting for pre-specified baseline variables, as detailed in the 'statistical analyses' section.

For peer review only

SUMMARY AND CONCLUSION

TXA is a widely available and inexpensive antifibrinolytic drug, which has the potential of reducing neurotoxicity and neuroinflammation in SICH. However, it is also possible that it may potentiate the risk of ischaemic events. The TICH-2 MRI sub-study will assess the effectiveness of TXA in reducing inflammatory response, while also determining whether there is an increased risk of ischaemic events following its administration. This will inform future studies of TXA and, if found to be effective and safe, have an important impact in clinical practice.

AUTHORS' CONTRIBUTION

RAD, KF, PSM, IR, DW, RAS, TE, PMB and NS designed the study and were co-applicants for funding. SP designed and will perform the image analyses. KF designed and will perform the statistical analyses. RAD, SP, ZKL, KF and NS drafted the manuscript. All authors reviewed and commented on the manuscript.

COMPETING INTERESTS

The authors have no conflicts of interest to disclose with respect to the research, authorship and publication of this article.

For peer review only

REFERENCES

1 McCormack PL. Tranexamic Acid: A review of its use in the treatment of hyperfibrinolysis. *Drugs* 2012;**72**:585–617.

2 Davis SM, Broderick J, Hennerici M, *et al.* Hematoma growth is a determinant of mortality and poor outcome after intracerebral hemorrhage. *Neurology* 2006;**66**:1175–81.

3 Sprigg N, Robson K, Bath PM, *et al.* Intravenous tranexamic acid for hyperacute primary intracerebral hemorrhage: Protocol for a randomized, placebo-controlled trial. *Int J stroke* 2016;**11**:717–23.

4 Prabhakaran S, Gupta R, Ouyang B, *et al.* Acute brain infarcts after spontaneous intracerebral hemorrhage: A diffusion-weighted imaging study. *Stroke* 2010;**41**:89–94.

5 Menon RS, Burgess RE, Wing JJ, *et al.* Predictors of highly prevalent brain ischemia in intracerebral hemorrhage. *Ann Neurol* 2012;**71**:199–205.

6 Gregoire SM, Charidimou A, Gadapa N, *et al.* Acute ischaemic brain lesions in intracerebral haemorrhage: Multicentre cross-sectional magnetic resonance imaging study. *Brain* 2011;**134**:2376–86.

7 Kang DW, Han MK, Kim HJ, *et al.* New ischemic lesions coexisting with acute intracerebral hemorrhage. *Neurology* 2012;**79**:848–55.

8 Garg RK, Liebling SM, Maas MB, *et al.* Blood pressure reduction, decreased diffusion on MRI, and outcomes after intracerebral hemorrhage. *Stroke* 2012;**43**:67–71.

9 Wardlaw JM, Smith EE, Biessels GJ, *et al.* Neuroimaging standards for research into small vessel disease and its contribution to ageing and neurodegeneration. *Lancet Neurol* 2013;**12**:822–38.

10 Schreiber S, Bueche CZ, Garz C, *et al.* Blood brain barrier breakdown as the

For peer review only

starting point of cerebral small vessel disease? - New insights from a rat model. *Exp Transl Stroke Med* 2013;**5**:4.

11 Baharoglu MI, Germans MR, Rinkel GJE, *et al.* Antifibrinolytic therapy for aneurysmal subarachnoid haemorrhage. *Cochrane database Syst Rev* 2013.

12 Ross J, Al-Shahi Salman R. The frequency of thrombotic events among adults given antifibrinolytic drugs for spontaneous bleeding: Systematic review and meta-analysis of observational studies and randomized trials. *Curr Drug Saf* 2012;**7**:44–54.

13 Perel P, Al-Shahi Salman R, Kawahara T, *et al.* CRASH-2 (Clinical Randomisation of an Antifibrinolytic in Significant Haemorrhage) intracranial bleeding study: the effect of tranexamic acid in traumatic brain injury--a nested randomised, placebo-controlled trial. *Health Technol Assess* 2012;**16**.

14 CRASH-2 Collaborators. Effect of tranexamic acid in traumatic brain injury: a nested randomised, placebo controlled trial (CRASH-2 Intracranial Bleeding Study). *BMJ* 2011;**343**:d3795.

15 Roberts I, Shakur H, Ker K, *et al.* Antifibrinolytic drugs for acute traumatic injury. *Cochrane database Syst Rev* 2011;**12**:CD004896.

16 Lo EH, Wang X, Cuzner ML. Extracellular proteolysis in brain injury and inflammation: Role for plasminogen activators and matrix metalloproteinases. *J Neurosci Res* 2002;**69**:1–9.

17 Chen ZL, Strickland S. Neuronal death in the hippocampus is promoted by plasmin-catalyzed degradation of laminin. *Cell* 1997;**91**:917–25.

18 Endo A, Nagai N, Urano T, *et al.* Proteolysis of neuronal cell adhesion molecule by the tissue plasminogen activator-plasmin system after kainate injection in the mouse hippocampus. *Neurosci Res* 1999;**33**:1–8.

19 Later AFL, Sitniakowsky LS, van Hilten JA, *et al.* Antifibrinolytics attenuate inflammatory gene expression after cardiac surgery. *J Thorac Cardiovasc Surg*

For peer review only

2013;**145**:1611–1616.e4.

20 Leira R, Dávalos A, Silva Y, *et al.* Early neurologic deterioration in intracerebral hemorrhage: Predictors and associated factors. *Neurology* 2004;**63**:461–7.

21 Fu Y, Hao J, Zhang N, *et al.* Fingolimod for the treatment of intracerebral hemorrhage: A 2-arm proof-of-concept study. *JAMA Neurol* 2014;**71**:1–10.

22 Li N, Liu Y., Ma L, *et al.* Association of molecular markers with perihematomal edema and clinical outcome in intracerebral hemorrhage. *Stroke* 2013;**44**:658–63.

23 Venkatasubramanian C, Mlynash M, Finley-Caulfield A, *et al.* Natural history of perihematomal edema following intracerebral hemorrhage measured by serial magnetic resonance imaging. *Stroke* 2011;**42**:73–80.

24 Butcher KS, Baird T, MacGregor L, *et al.* Perihematomal edema in primary intracerebral hemorrhage is plasma derived. *Stroke* 2004;**35**:1879–85.

25 Carhuapoma JR, Barker PB, Hanley DF, *et al.* Human brain hemorrhage: Quantification of perihematoma edema by use of diffusion-weighted MR imaging. *Am J Neuroradiol* 2002;**23**:1322–6.

26 Kidwell CS, Saver JL, Mattiello J, *et al.* Diffusion-perfusion MR evaluation of perihematomal injury in hyperacute intracerebral hemorrhage. *Neurology* 2001;**57**:1611–7.

27 Pszczolkowski S, Gallagher RG, Law ZK, *et al.* Automatic Quantification of Haematoma and Surrounding Oedema in MRI of Acute Spontaneous Intracerebral Haemorrhage: Preliminary Results for the TICH-2 MRI Sub-study. In: *Proc. ISMRM*. 2017.

28 Wahlund LO, Barkhof F, Fazekas F, *et al.* A new rating scale for age-related white matter changes applicable to MRI and CT. *Stroke* 2001;**6**:1318–23.

29 Gregoire SM, Chaudhary UJ, Brown MM, *et al.* The Microbleed Anatomical Rating Scale (MARS): Reliability of a tool to map brain microbleeds. *Neurology*

For peer review only

2009;**73**:1759–66.

30 Sprigg N, Renton CJ, Dineen RA, *et al.* Tranexamic acid for spontaneous intracerebral hemorrhage: A randomized controlled pilot trial (ISRCTN50867461). *J Stroke Cerebrovasc Dis* 2014;**23**:1312–8.

31 Law ZK, Meretoja A, Engelter ST, *et al.* Treatment of intracerebral haemorrhage with tranexamic acid - A review of current evidence and ongoing trials. *Eur Stroke J* 2017;**2**:13–22.

For peer review only

Figure 1 – TICH-2 MRI substudy flowchart. Details of participant flow for the TICH-2 trial, including the schedule for randomisation, treatment, cranial CT, clinical assessments and non-MRI follow-up assessments have been described in Sprigg et al [3].

Figure 2 – Summary of image processing and outputs in the TICH-2 MRI substudy.

Figure 3 - Measurement of perihaematomal oedema (PHO) volume and apparent diffusion coefficient (ADC). (a) Axial FLAIR image in native T1 space showing the PHO delineation from which the PHO volume can be derived. (b) ADC map in native T1 space. PHO ADC values are calculated from the voxels within the PHO delineation in the ADC map.

For peer review only

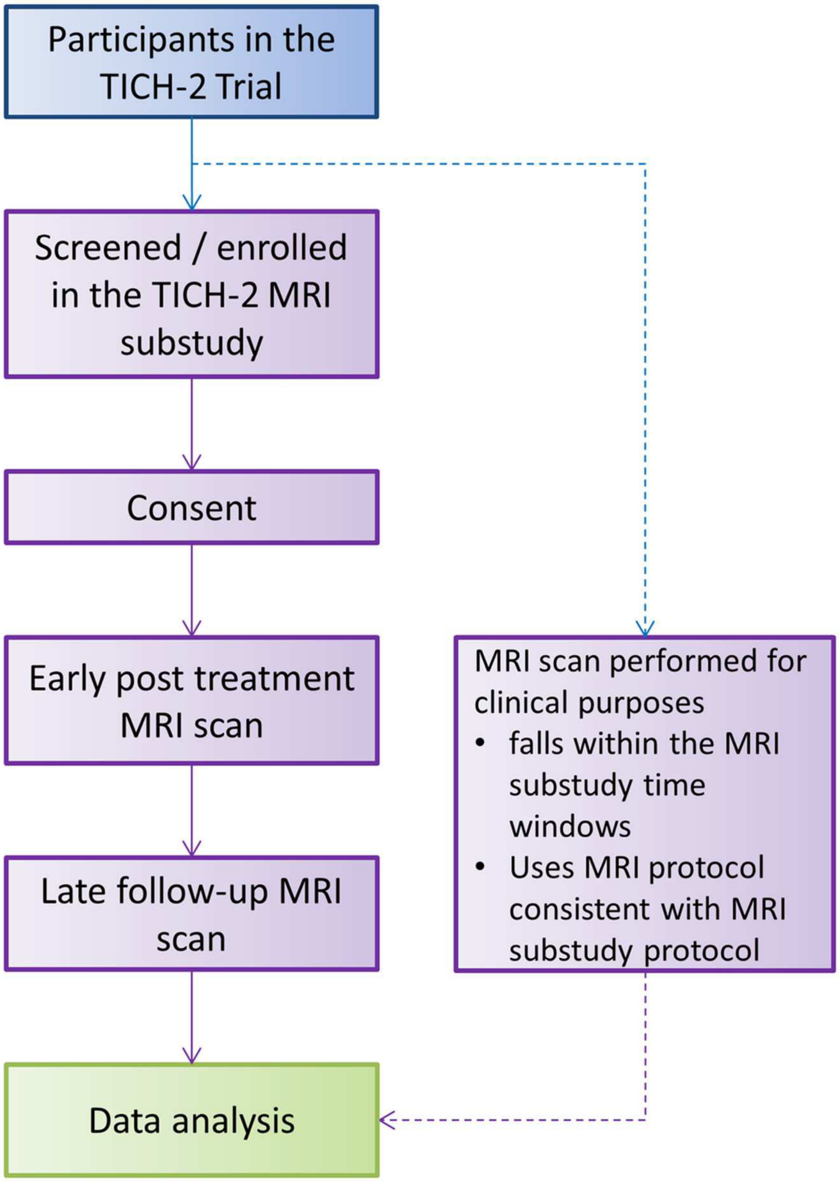

TICH-2 MRI substudy flowchart. Details of participant flow for the TICH-2 trial, including the schedule for randomisation, treatment, cranial CT, clinical assessments and non-MRI follow-up assessments have been described in Sprigg et al [3].

42x59mm (600 x 600 DPI)

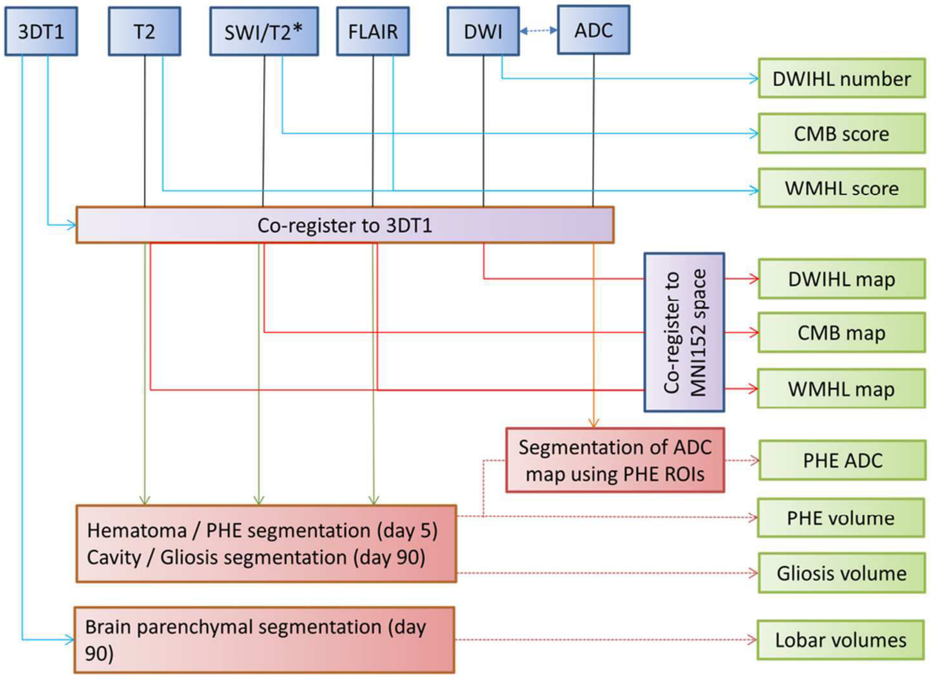

Summary of image processing and outputs in the TICH-2 MRI substudy.

47x35mm (600 x 600 DPI)

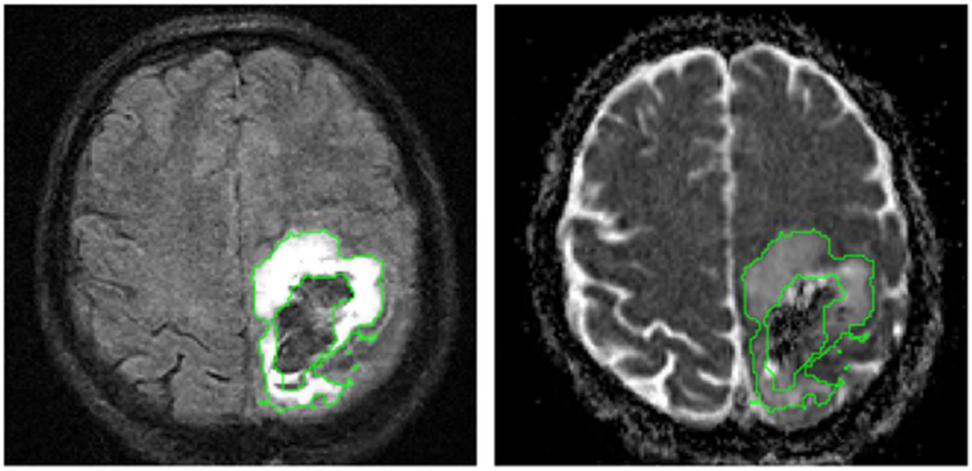

(a) (b)

Measurement of perihaematomal oedema (PHO) volume and apparent diffusion coefficient (ADC). (a) Axial FLAIR image in native T1 space showing the PHO delineation from which the PHO volume can be derived. (b) ADC map in native T1 space. PHO ADC values are calculated from the voxels within the PHO delineation in the ADC map.

24x13mm (600 x 600 DPI)

For peer review only

Supplementary information: Brief summary of the TICH-2 Trial design with list of inclusion and exclusion criteria*

TICH-2 is a phase III prospective double-blind randomized placebo-controlled trial that aims to test the hypothesis that intravenous tranexamic acid given within 8 h of SICH reduces death or dependency. 2000 participants within 8 hours of SICH will be randomized to receive either intravenous tranexamic acid 1 g 10 min bolus followed by 1 g 8 h infusion, or placebo. The primary outcome is death or dependency measured by ordinal shift analysis of the 7 level mRS at
day 90. Secondary outcomes are neurological impairment at day 7 and disability, quality of life, cognition, and mood at day 90. Safety outcomes are death, serious adverse events, thromboembolic events, and seizures. Cost outcomes are length of stay in hospital, readmission, and institutionalization.

Inclusion criteria:

- Adults with acute SICH within 8 h of stroke symptom onset or time last seen well.

Exclusion criteria:

- Patients with ICH secondary to anticoagulation, thrombolysis or known underlying structural abnormality such as arteriovenous malformation, aneurysm, tumour, or venous thrombosis. An underlying structural abnormality does not need to be excluded before enrolment, but where known, patients should not be recruited.
- Contraindication to tranexamic acid.
- Premorbid dependency (mRS>4).
- Concurrent participation in another drug or device trial. Participants enrolled in TICH-2 may be enrolled into the RESTART trial18 after 21 days.
- Pre-stroke life expectancy <3 months (e.g. advanced metastatic cancer).
- Coma—Glasgow coma scale <5.
- ICH was secondary to trauma.
- Women of childbearing potential, pregnant, or breastfeeding at randomization.
- Geographical or other factors that prohibit follow-up at 90 days, e.g. no fixed address or telephone contact number, or overseas visitor.

* Sprigg N, Robson K, Bath PM, *et al.* Intravenous tranexamic acid for hyperacute primary intracerebral hemorrhage: Protocol for a randomized, placebo-controlled trial. *Int J stroke* 2016;**11**:717–23

For peer review only
